# Comparison of miRNA and mRNA Expression in Sika Deer Testes With Age

**DOI:** 10.3389/fvets.2022.854503

**Published:** 2022-04-05

**Authors:** Boyin Jia, Linlin Zhang, Fuquan Ma, Xue Wang, Jianming Li, Naichao Diao, Xue Leng, Kun Shi, Fanli Zeng, Ying Zong, Fei Liu, Qinglong Gong, Ruopeng Cai, Fuhe Yang, Rui Du, Zhiguang Chang

**Affiliations:** ^1^College of Animal Medicine/College of Animal Science and Technology, Jilin Agricultural University, Changchun, China; ^2^Laboratory of Production and Product Application of Sika Deer of Jilin Province, Jilin Agricultural University, Changchun, China; ^3^College of Chinese Medicine Materials, Jilin Agricultural University, Changchun, China; ^4^Institute of Wild Economic Animals and Plants and State Key Laboratory for Molecular Biology of Special Economical Animals, Chinese Academy of Agricultural Sciences, Changchun, China; ^5^The Seventh Affiliated Hospital, Sun Yat-sen University, Shenzhen, China

**Keywords:** sika deer, testis development, spermatogenesis, mRNA, miRNA

## Abstract

To elucidate the complex physiological process of testis development and spermatogenesis in Sika deer, this study evaluated the changes of miRNA and mRNA profiles in the four developmental stages of testis in the juvenile (1-year-old), adolescence (3-year-old), adult (5-year-old), and aged (10-year-old) stages. The results showed that a total of 198 mature, 66 novel miRNAs, and 23,558 differentially expressed (DE) unigenes were obtained; 14,918 (8,413 up and 6,505 down), 4,988 (2,453 up and 2,535 down), and 5,681 (2,929 up and 2,752 down) DE unigenes, as well as 88 (43 up and 45 down), 102 (44 up and 58 down), and 54 (18 up and 36 down) DE miRNAs were identified in 3- vs. 1-, 5- vs. 3-, and 10- vs. 5-year-old testes, respectively. By integrating miRNA and mRNA expression profiles, we predicted 10,790 mRNA–mRNA and 69,883 miRNA–mRNA interaction sites. The target genes were enriched by GO and KEGG pathways to obtain DE mRNA (IGF1R, ALKBH5, Piwil, HIF1A, BRDT, etc.) and DE miRNA (miR-140, miR-145, miR-7, miR-26a, etc.), which play an important role in testis development and spermatogenesis. The data show that DE miRNAs could regulate testis developmental and spermatogenesis through signaling pathways, including the MAPK signaling pathway, p53 signaling pathway, PI3K-Akt signaling pathway, Hippo signaling pathway, etc. miR-140 was confirmed to directly target mutant IGF1R-3′UTR by the Luciferase reporter assays. This study provides a useful resource for future studies on the role of miRNA regulation in testis development and spermatogenesis.

## Introduction

Testis development and spermatogenesis are the major processes in male reproduction. Germ cells are the direct participants in spermatogenesis, which is the key in reproduction (1). Spermatogenesis is a complex process of cellular divisions and developmental changes in testicular seminiferous tubules (2). However, there are few studies on gene regulation of testis development in Cervidae family. It was only reported that MT1, MT2, VEGF, INSL3, LGR8, aFGF, bFGF, IGF-1, IGF-2, TGF alpha genes of roe deer, and steroidogenic enzymes (P450scc, P450c17, 3betaHSD, and P450arom) of Sika Deer were involved in the regulation of testis development and spermatogenesis (3–7). In addition to mRNA encoding proteins, many ncRNAs are also involved in regulation, including miRNAs.

miRNA specifically binds to the 3′UTR sequences of mRNA to degrade target genes or inhibit the translation of target genes, thereby regulating gene expression and participating in biological processes (8). miRNA presents a major effect during the testis development and spermatogenesis (9). miRNA is involved in three distinctive phases of spermatogenesis, which include mitosis of spermatogonia, meiosis of spermatocytes, and maturation of spermatids (9). For example, miR-34c and miR-221 regulate spermatogonial stem cells self-renewal by target gene Nanos2 and c-Kit (10, 11); miR-17-92 cluster regulates spermatogonial differentiation by target gene Stat3, Socs3, Bim, and c-Kit (12); miR-34b/c regulates meiosis of spermatocytes by target gene FoxJ2 (13); miR-34 and miR-122 regulate sperm development by target gene GSK3a and TNP2 (14, 15). However, there are no reports about miRNAs regulating spermatogenesis in deer. With the continuous in-depth study of miRNAs regulating spermatogenesis, it is important to understand the mechanism of deer miRNAs regulating spermatogenesis through target genes, which in turn affects male deer reproduction.

The expression of miRNA in the testis is species-specific and stage-specific. So far, many miRNAs have been identified in different species, but there are obvious differences in these miRNAs. For example, Yang et al. used miRNA microarray to analyze the testicular tissues of rhesus monkey and human. The results showed that 26.4% of miRNAs were differentially expressed in rhesus monkeys (such as mir-493-3p, mir-376b, miR-222, etc.), and 31.3% of miRNAs were differentially expressed in humans (such as miR-181c, let-7e, mir-219, etc.) (16). In addition, miRNA expression changes with testis development and spermatogenesis. Gao et al. found that 223 miRANs were differentially expressed in bovine testes at neonatal (3 days after birth) and mature (13 months) stages by RNA-seq (17). Bai et al. found that 137, 106, and 28 miRNAs were differentially expressed in sheep testes in 2 vs. 6, 6 vs. 12, and 2 vs. 12 months, respectively (18). Ran et al. found that 93, 104, and 122 miRNAs were differentially expressed in pig testes in 90-dpc (days post coitus) vs. 60-dpc, 30 days vs. 90-dpc, and 180 days vs. 30-day, respectively (19). However, there is still a lack of research on the expression patterns and mechanisms of miRNAs at different developmental stages in the testis of deer family.

There were no reports on types of germ cells in different stages in Cervidae, but Sertoli cells, primary spermatocytes, secondary spermatocytes, round spermatids and long spermatids were observed in the juvenile bovine testis closely related to Cervidae. All classes of germ cells were observed in the adolescent cattle testis, although the numbers of germ cells were small. Complete spermatogenesis was observed on adult stage and the number of sperm was large (20). Therefore, in this study, we selected the four developmental stages (juvenile, adolescent, adult, and aged) of the Sika Deer testis as the research object, and used Illumina sequencing technology to establish a comprehensive mRNA and miRNA expression profile of testis of sika deer in the whole life stage for the first time. We further constructed a miRNA–mRNA interaction network related to Sika Deer testis development and spermatogenesis. The results of this study will help to determine the molecular markers that affect the reproductive efficiency of male Sika Deer and provide a reference for finding molecular markers that regulate the reproductive ability of male Sika Deer.

## Materials and Methods

### Tissues Collection

In this study, 12 Sika Deer were divided into four groups, namely the juvenile group: 1-year-old (Tst_1), the adolescence group: 3 years old (Tst_2), the adult group: 5 years old (Tst_3), and the aged group: 10 years old (Tst_4). After manual slaughter, testicular tissues were taken, frozen, and stored in liquid nitrogen until RNA extraction. All operations of Sika Deer in this study strictly followed the guidelines approved by the Ethics Committee of Jilin Agricultural University.

### mRNA and miRNA Sequencing and Data Analysis

Total RNA was isolated from testis with TRIzol reagent. The Agilent 2100 bioanalyzer and NanoDrop 2000 were used to measure the quality, concentration, and integrity of RNA. Total RNA pool was collected from testes of three individuals to construct an mRNA library or a small RNA library for each growth period. According to the method we described earlier, mRNA library and small RNA library were generated and sequenced on an Illumina HiSeq2500 platform (21). The unigenes (the longest transcript of each gene) with *P*_*adj*_ < 0.05 and |log_2_(fold change)| >0 were taken as DE unigenes (22). The DE miRNAs were identified by threshold [*q* < 0.005 and |log_2_(fold change) | >1].

### miRNA–mRNA Network Integration

miRanda and TargetScan were used to predict the target mRNAs of known and novel miRNAs. The putative target mRNAs were crossed with DE miRNAs in Tst_2 vs. Tst_1, Tst_3 vs. Tst_2, Tst_4 vs. Tst_3, respectively. Then the Pearson correlation coefficient was used to determine the candidate target mRNA the expression level of which was negatively correlated with miRNA. Finally, the regulatory networks of DE miRNA and target mRNA were modeled in Cytoscape 3.5.1.

### GO and KEGG Pathway Analyses

All the DE mRNAs and DE miRNA target mRNAs were analyzed using the GO (http://geneontology.org) and KEGG (www.genome.jp/kegg) databases. The GO enrichment was used to analyze the functions of mRNAs. The *p*-value of GO terms < 0.05 was significantly enriched. Similarly, the KEGG enrichment was used to analyze the pathways in which the mRNAs were involved. The *p*-value of KEGG terms < 0.05 was significantly enriched.

### Real-Time Fluorescent Quantitative PCR

Nine DE mRNAs (PPP2R4, Calm1, SLC7A5, DST, GSTM1, TIMP2, USF2, ITPKB, and GDI2) and nine DE miRNAs (miR-7, miR-124a, miR-145, let-7b, miR-214, miR-196a, miR-26a, miR-125a, and miR-574) were selected for analysis of differential expression levels. For each sample, mRNAs and miRNAs were reverse-transcribed using PrimeScript™ RT reagent Kit with gDNA Eraser (Takara, Shiga, Japan) and miScript II RT Kit (Qiagen, Hilden, Germany), respectively. Q-PCR analyses on the mRNAs and miRNAs were confirmed using TB Green® Premix Ex Taq™ II (Takara) and miScript SYBR Green PCR Kit (Qiagen) in the ABI Prism 7900 System (Ambion, Carlsbad, CA, USA). GAPDH and U6 snRNA were selected as internal control of mRNA and miRNA, respectively. The 2^−ΔΔ^CT method was used to evaluate relative expression levels between surveys.

### Dual-Luciferase Reporter Assays

The 3′-UTR fragments of IGF1R containing the wild-type (WT-IGF1R) or mutant (Mut-IGF1R) were cloned into the psiCHECK-2 vector. The vectors were co-transfected with miR-140 mimic into HEK-293T cells by Lipofectamine 2000 transfection reagent (Invitrogen, Carlsbad, CA, USA). Luciferase activities were measured using a Dual-Luciferase Reporter Assay System (Promega Corporation, Madison, WI, USA) after transfection for 48 h.

### Data Availability

All the sequencing data of this study have been submitted to the NCBI Gene Expression Omnibus. The accession number was GSE188370.

## Results

### Overview of mRNA Library

A total of 34,792,106; 34,728,704; 27,299,920; and 29,437,362 raw reads were generated in the Tst_1, Tst_2, Tst_3, and Tst_4 libraries, respectively. Removing the adaptors and low-quality sequences, a total of 34,199,251 (98.30%), 34,183,418 (98.43%), 26,940,428 (98.68%), and 29,030,291 (98.62%) clean reads were obtained for further analysis (Supplementary File 1). A total of 56,698 (59.26%), 57,243 (57.91%), 58,169 (60.69%), 55,461 (58.72%) unigenes which FPKM >0.3 were obtained (Supplementary File 2). The results showed that as the testis matured, the number of unigenes increased significantly. On the contrary, for testicular aging, the number of unigenes decreases significantly.

### DE Unigenes and Functional Annotation Analysis

**Figure 1:**
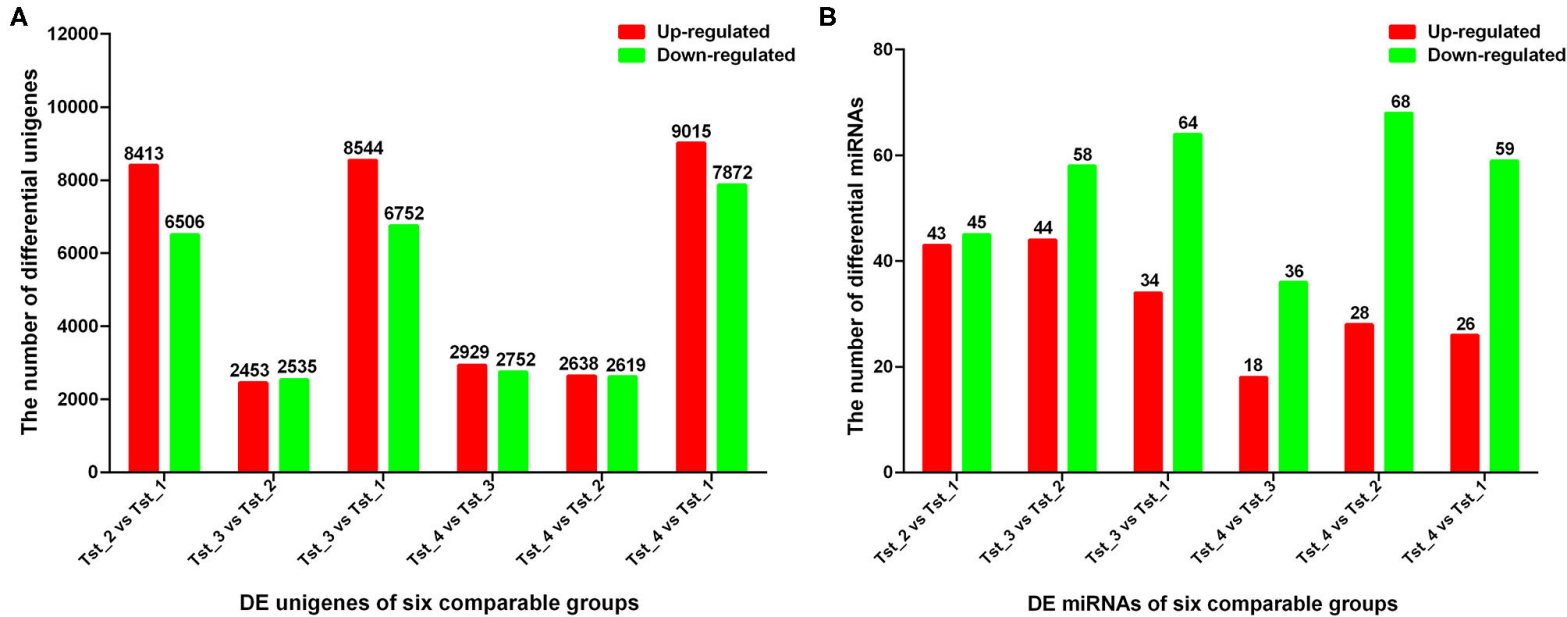
Statistics for DE unigenes and miRNAs in each comparable group. **(A)** *q* value < 0.005 and |log2 (*fold change*)| >1 were used as thresholds of significance for DE unigenes. **(B)** *q* value < 0.01 and |*log*2 (*fold change*)| >1 were used as thresholds of significance for DE miRNAs.

In the present study, a total of 23,558 DE unigenes among 80,133 expressed unigenes existed in the testis of Tst_1, Tst_2, Tst_3, and Tst_4 groups by calculating the |log_2_(fold change) | >0 and *p*_*Adj*_ < 0.05 as the cut off. Among them, 8,413 up- and 6,505 downregulated, 2,453 up- and 2,535 downregulated, 8,544 up- and 6,752 downregulated, 2,929 up- and 2,752 downregulated, 2,638 up- and 2,619 downregulated, and 9,015 up- and 7,872 downregulated unigenes were detected in Tst_2 vs. Tst_1, Tst_3 vs. Tst_2, Tst_3 vs. Tst_1, Tst_4 vs. Tst_3, Tst_4 vs. Tst_2, and Tst_4 vs. Tst_1, respectively (Figure 1A, Supplementary File 3).

In GO enrichment analysis, 112, 263, and 205 GO BP terms were found from Tst_2 vs. Tst_1, Tst_3 vs. Tst_2, and Tst_4 vs. Tst_3, respectively (*p* < 0.05; Supplementary File 4). In Tst_2 vs. Tst_1, the most enriched GO BP terms were mainly involved in the synthesis and metabolism of sugar, protein, and lipids, such as: carbohydrate derivative biosynthetic process, cellular carbohydrate biosynthetic process, cellular carbohydrate metabolic process, cellular lipid metabolic process, cellular polysaccharide biosynthetic process, cellular polysaccharide metabolic process, and cellular protein metabolic process. In Tst_3 vs. Tst_2, the most enriched GO BP terms were mainly involved in the localization and transport of cellular and protein, such as cellular localization, intracellular transport, intracellular protein transport, and cellular protein localization. In Tst_4 vs. Tst_3, the most enriched GO BP terms were mainly involved in the microtubule, such as microtubule-based process and microtubule-based movement. The top 20 enriched GO terms were shown in Supplementary Figure 1. In pathway analysis, 77, 61, and 75 enriched pathways were detected in Tst_2 vs. Tst_1, Tst_3 vs. Tst_2, and Tst_4 vs. Tst_3, respectively (p < 0.05; Supplementary File 5). In Tst_2 vs. Tst_1, the most significant pathway was enriched in the Hedgehog signaling pathway, Cell adhesion molecules (CAMs), MAPK signaling pathway, insulin signaling pathway, estrogen signaling pathway, and glycerophospholipid metabolism. In Tst_3 vs. Tst_2, the most significant pathway was enriched in the phagosome, hedgehog signaling pathway, and glycerophospholipid metabolism. In Tst_4 vs. Tst_3, the most significant pathway was enriched in the phagosome, thyroid hormone signaling pathway, and glycerophospholipid metabolism. The top 20 significantly enriched pathways are shown in Supplementary Figure 2.

### Interaction Network Analysis of DE Unigenes

**Figure 2:**
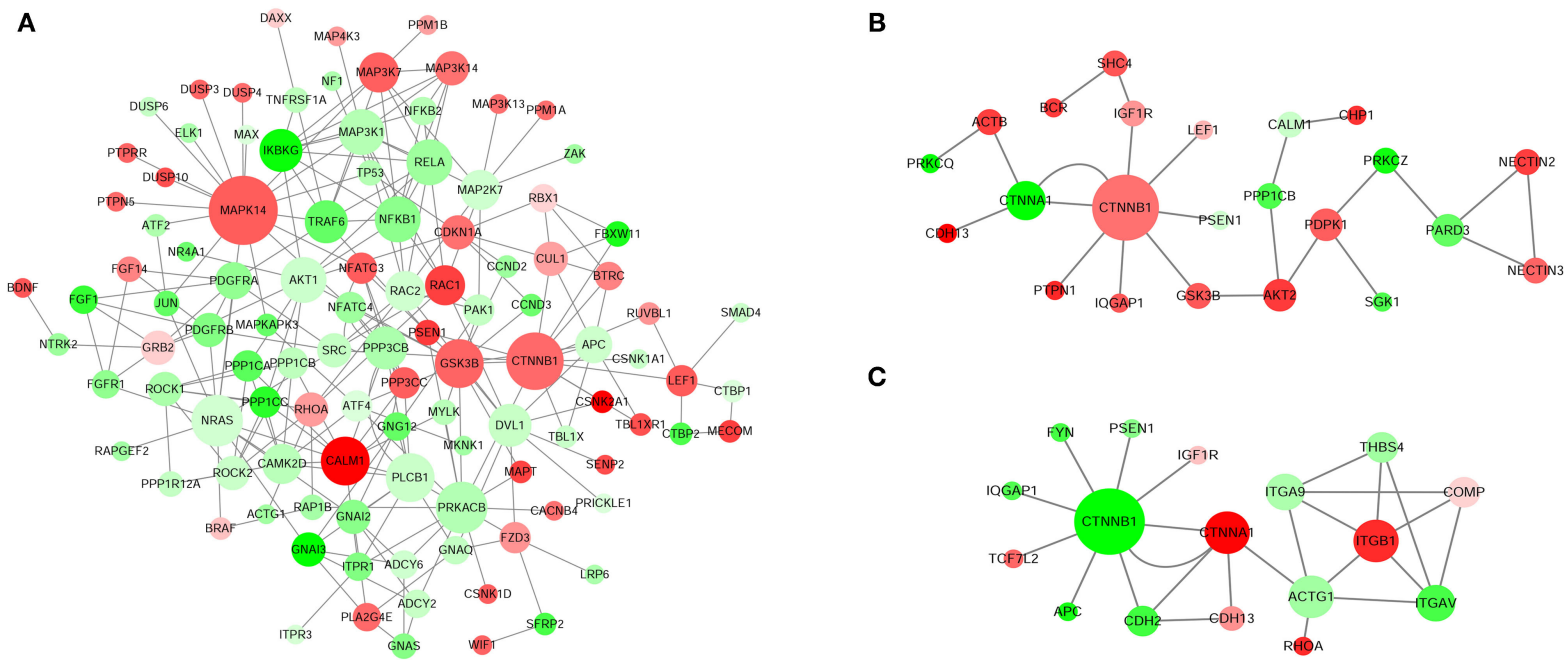
Interaction networks of top DE unigenes in each comparable group. **(A)** Tst_2 vs. Tst_1. **(B)** Tst_3 vs. Tst_2. **(C)** Tst_4 vs. Tst_3. The up-regulated unigenes were displayed as red circles, and the down-regulated unigenes were displayed as green circles.

A total of 7,658, 1,533, and 1,599 mRNA–mRNA pairs in Tst_2 vs. Tst_1, Tst_3 vs. Tst_2, Tst_4 vs. Tst_3 contrasts were predicted in our study. In addition, DAVID was also used to examine which mRNA-mRNA interaction networks were enriched (Supplementary File 6). In Tst_2 vs. Tst_1, we detected that most interaction networks were involved in the MAPK signaling pathway, Wnt signaling pathway, oxytocin signaling pathway, hedgehog signaling pathway (Figure 2A, Table 1), and, especially, members of Wnt signaling pathway, the GSK3B, PRKACB, CTNNB1, and PLCB1, were the core of this network. In Tst_3 vs. Tst_2, we detected that most interaction networks were involved in the adherens junction, insulin signaling pathway, intracellular signal transduction, calcium (Figure 2B, Table 1), and especially, insulin signaling pathway, including SHC4, PTPN1, GSK3B, PDPK1, PRKCZ, PPP1CB, AKT2, and CALM1. In Tst_4 vs. Tst_3, we detected that most interaction networks were involved in arrhythmogenic right ventricular cardiomyopathy, adherens junction, calcium, and positive regulation of apoptotic process (Figure 2C, Table 1). The result indicated that the pathways of the adherens junction and calcium might play important roles both in Tst_3 vs. Tst_2 and Tst_4 vs. Tst_3. The members of the adherens junction pathway CTNNA1 and CTNNB1 were the core of these networks.

**Table 1:** Summary of the represented networks generated by pathway analysis.

**Data**	**Molecules in networks**	**Score**	***p*** **-Value**	**Top functions**
Tst_2 vs. Tst_1	AKT1, GRB2, ATF2, PTPRR, ZAK, FGF1, ELK1, PPP3CB, PPP3CC, DUSP10, MECOM, RAC2, RAC1, IKBKG, PRKACB, MAP3K7, PDGFRB, DUSP4, PDGFRA, DAXX, DUSP3, PLA2G4E, DUSP6, TNFRSF1A, PPM1A, PPM1B, CACNB4, MAPKAPK3, TRAF6, RAPGEF2, MAPT, TP53, ATF4, MAX, RELA, RAP1B, NRAS, PAK1, MKNK1, MAP2K7, MAP4K3, NTRK2, JUN, MAP3K1, BDNF, NFATC3, BRAF, GNG12, MAPK14, NFKB1, NFKB2, NR4A1, FGF14, NF1, MAP3K13, PTPN5, MAP3K14, FGFR1	108	0.003	MAPK signaling pathway
	SMAD4, GSK3B, CAMK2D, CTBP2, CTBP1, ROCK2, LEF1, CUL1, PRICKLE1, PSEN1, LRP6, CCND3, PPP3CB, PPP3CC, CCND2, WIF1, RUVBL1, DVL1, RAC2, TBL1X, RAC1, BTRC, PRKACB, MAP3K7, FZD3, JUN, CSNK2A1, FBXW11, CSNK1A1, NFATC3, SENP2, RHOA, NFATC4, RBX1, SFRP2, APC, TBL1XR1, CTNNB1, PLCB1, TP53		0.003	Wnt signaling pathway
	CDKN1A, CAMK2D, ROCK1, SRC, ROCK2, ITPR1, GNAI3, ITPR3, ADCY2, ELK1, ADCY6, ACTG1, MYLK, GNAI2, PPP1CB, PPP1CC, NRAS, PPP3CB, PPP3CC, PRKACB, JUN, PPP1R12A, PLA2G4E, NFATC3, RHOA, PPP1CA, NFATC4, CACNB4, GNAQ, GNAS, CALM1, PLCB1		0.023	Oxytocin signaling pathway
	GSK3B, CSNK1A1, FBXW11, CSNK1D, BTRC, PRKACB		0.003	Hedgehog signaling pathway
Tst_3 vs. Tst_2	PTPN1, PARD3, LEF1, CTNNA1, CTNNB1, IQGAP1, NECTIN3, ACTB, NECTIN2, IGF1R	23	0.003	Adherens junction
	SHC4, PTPN1, GSK3B, PDPK1, PRKCZ, PPP1CB, AKT2, CALM1		0.031	Insulin signaling pathway
	PDPK1, PSEN1, PRKCZ, BCR, AKT2, PRKCQ, SGK1		0.035	Intracellular signal transduction
	CDH13, CHP1, CALM1		0.033	Calcium
Tst_4 vs. Tst_3	ITGB1, TCF7L2, CDH2, CTNNA1, CTNNB1, ITGAV, ITGA9, ACTG1	17	0.044	Arrhythmogenic right ventricular cardiomyopathy
	TCF7L2, CTNNA1, CTNNB1, FYN, IQGAP1, RHOA, IGF1R, ACTG1		0.011	Adherens junction
	THBS4, COMP, CDH2, CDH13, ITGAV		0.008	Calcium
	APC, CTNNB1, PSEN1		0.028	Positive regulation of apoptotic process

### Overview of Small RNA Library

Sequencing of four libraries produced 11,297,281; 13,817,801; 12,142,852; and 10,566,461 raw reads, respectively. A total of 11,057,680 (97.88%), 13,457,309 (97.39%), 11,808,585 (97.25%), and 10,353,783 (97.99%) clean reads were obtained after removing the contaminant and adaptor reads (Supplementary File 7). These sequences were further mapped to the bovine reference genome (Supplementary File 8). In addition, an average of 1.7% of clean reads were mapped to miRNA, rRNA, tRNA, snRNA, and snoRNA (Supplementary File 9). A total of 183, 137, 120, and 100 known miRNAs and 45, 26, 32, and 27 novel miRNAs were identified from four development stages, respectively (Supplementary File 10). Among them, 92, 17, and 32 miRNAs were co-expressed in four stages, three stages, and two stages, respectively. It was worth noting that 43, 6, 6, and 2 miRNAs were specifically expressed only in the juvenile, adolescence, adult, and the aged group, respectively. The length distributions of small RNA are shown in Supplementary Figure 3. The ranges of 20–24 nt small RNAs were the main sizes and accounted for at least 89.5% of the population in Tst_1, while Tst_2, Tst_3, and Tst_4 were mainly 25–32 nt in size.

### DE miRNAs and Their Target Genes Functional Annotation Analysis

**Figure 3:**
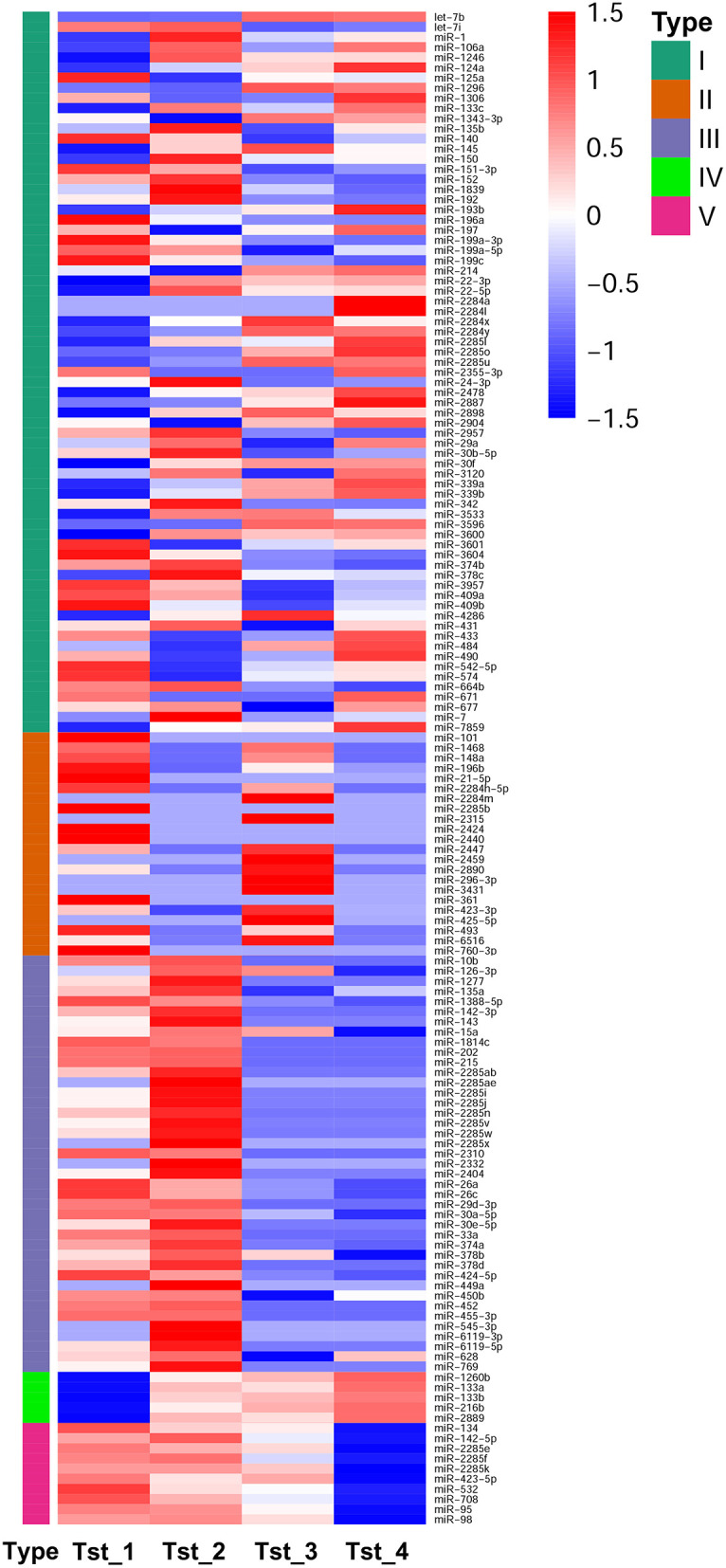
Hierarchical clustering of DE miRNAs.

According to DESeq analysis between the four libraries by calculating the log_2_-ratio with *q* < 0.005 and |log_2_(fold change)| >1 as the threshold, 88, 102, 98, 54, 96, and 85 miRNAs were different in Tst_2 vs. Tst_1, Tst_3 vs. Tst_2, Tst_3 vs. Tst_1, Tst_4 vs. Tst_3, Tst_4 vs. Tst_2, and Tst_4 vs. Tst_1, respectively (Supplementary File 11). In Tst_2 vs. Tst_1, 43 miRNAs were upregulated, and 45 miRNAs were downregulated. In Tst_3 vs. Tst_2, 44 miRNAs were upregulated, and 58 miRNAs were downregulated. In Tst_3 vs. Tst_1, 34 miRNAs were upregulated, and 64 miRNAs were downregulated. In Tst_4 vs. Tst_3, 18 miRNAs were upregulated, and 36 miRNAs were downregulated. In Tst_4 vs. Tst_2, 28 miRNAs were upregulated, and 68 miRNAs were downregulated. In Tst_4 vs. Tst_1, 26 miRNAs were upregulated, and 59 miRNAs were downregulated (Figure 1B, Supplementary File 11). Moreover, the hierarchical clusters of DE miRNAs between four stages indicated there were a large number of DE miRNA in the development of Sika Deer testis (Figure 3).

In order to identify the potential functional of miRNAs, miRanda was used to predict the target gene of DE miRNAs. In total, 36,571; 19,504; and 13,808 target genes of DE miRNAs were predicted in Tst_2 vs. Tst_1, Tst_3 vs. Tst_2, and Tst_4 vs. Tst_3, respectively. We further analyzed the function of target genes during the development of Sika Deer testis according to GO and KEGG analysis. There was no significant difference in the GO enrichment among four comparisons, mainly including substance and energy synthesis, protein modification, metabolic process, cell mitosis, etc. (Supplementary Figure 4). Similarly, there was no significant difference in the KEGG pathway enrichment among four comparisons, mainly including, PI3K-Akt signaling pathway, Focal adhesion, protein digestion and absorption, ECM-receptor interaction, etc. (Supplementary Figure 5).

### Identification of DE Unigenes Involved in Testis Development and Spermatogenesis and Their Interacting miRNAs

**Figure 4:**
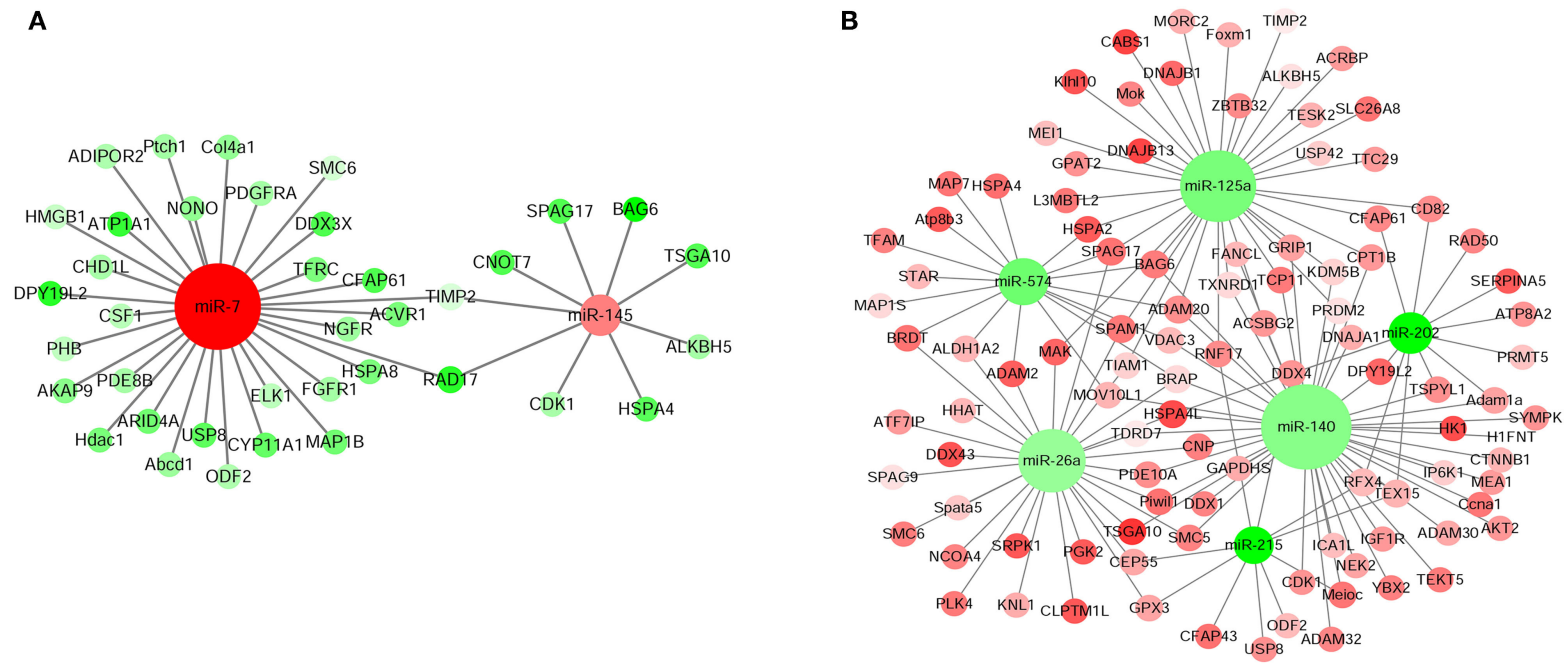
miRNA-mRNA interaction network related to testis development of Sika Deer. **(A)** Up-regulated miRNAs and down-regulated target genes related to testis development. **(B)** Down-regulated miRNAs and up-regulated target genes related to testis development. The up-regulated miRNAs or genes were displayed as red circles, and the down-regulated miRNAs or genes were displayed as green circles.

**Figure 5:**
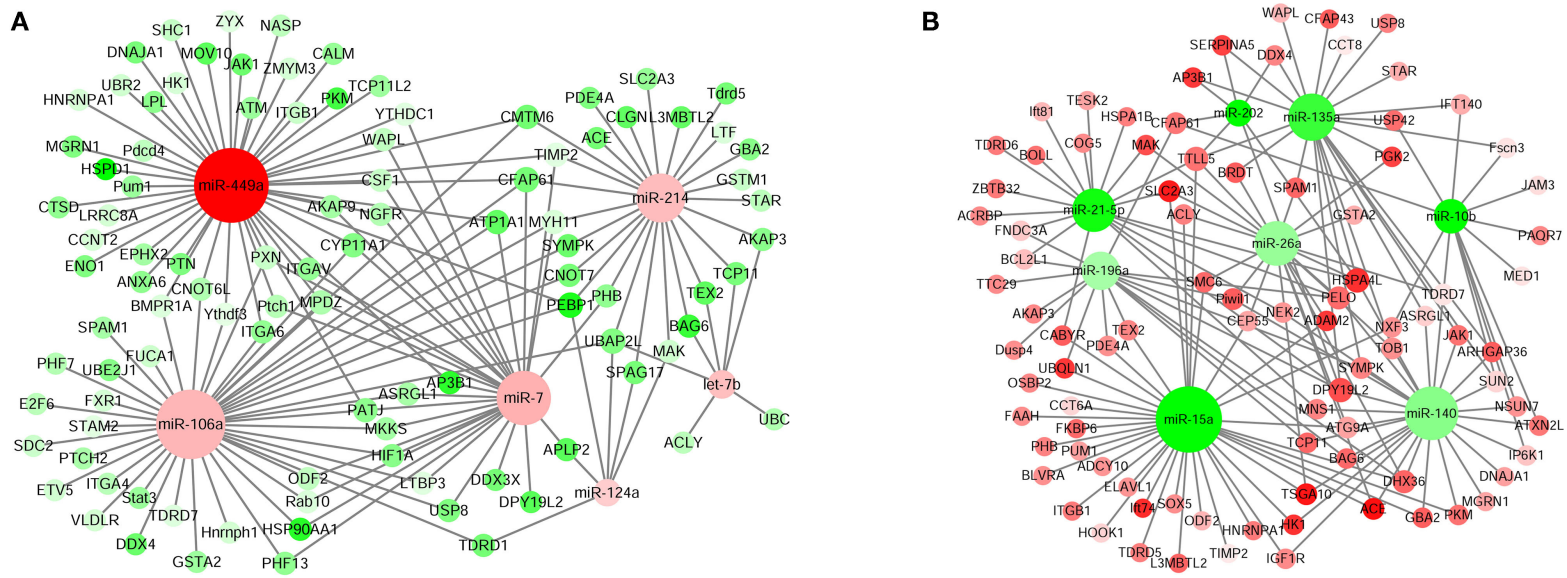
miRNA-mRNA interaction network related to spermatogenesis of Sika Deer. **(A)** Up-regulated miRNAs and down-regulated target genes related to spermatogenesis. **(B)** Down-regulated miRNAs and up-regulated target genes related to spermatogenesis. The up-regulated miRNAs or genes were displayed as red circle, and the down-regulated miRNAs or genes were displayed as green circles.

The interaction network of DE unigenes and DE miRNAs, which regulated the testis development and spermatogenesis of Sika Deer, was predicted by STRING website and visualized by Cytoscape 3.5.1. Integrating the miRNA-gene interaction network and previous reports, we found that there were 18 miRNAs involved in the regulation of testis development and spermatogenesis (Table 2). Among them, 189 pairs of miRNA-gene were negatively related to testis development, and 331 pairs of miRNA-gene were negatively related to spermatogenesis (Figures 4, 5). Figure 4A shows that miR-7 and miR-145 were upregulated which targeted HMGB1, HSPA8, CYP11A1, ALKBH5, etc., associated with testis development. In contrast, Figure 4B shows that miR-26a, miR-574, miR-140, miR-125a, miR-202, and miR-215 were downregulated, which targeted IGF1R, Piwil, StAR, TSPYL1, etc., associated with testis development. Similarly, Figure 5A shows that let-7b, miR-214, miR-124a, miR-106a, miR-449a, and miR-7 were upregulated, which targeted HIF1A, CSF1, TCP11, SPAG17, PEBP1, AKAP3, CNOT7, PHB, etc., associated with spermatogenesis. On the contrary, Figure 5B shows that miR-135a, miR-196a, miR-10b, miR-21-5p, miR-15a, miR-26a, miR-140, and miR-202 were downregulated, which targeted DPY19L2, HSPA4L, PELO, Piwil1, TSGA10, IP6K1, MNS1, BRDT, CEP55, SMC6, etc., associated with spermatogenesis. The above miRNAs contributed significantly to the regulation of mRNA expression during the testis development and spermatogenesis of Sika Deer.

**Table 2:** miRNAs identified in testis associated with testis development and spermatogenesis.

**miRNA ID**	**Testis development**	**miRNA ID**	**Spermatogenesis**
miR-145	Regulating the tight junctions of the epididymis by targeting Cldn10 (23)	let-7b	Regulating the glycolysis in asthenozoospermia by targeting AURKB (24)
miR-7a	Regulating the FSH and LH synthesis and secretion by pituitary prostaglandin and BMP4 signaling (25)	miR-214	Regulating the meiosis by targeting WD and WDTC1 (26)
miR-26a	Regulating the testis steroidogenesis by targeting FGF9 (27)	miR-124a	Regulating the proliferation of immature sertoli cells by targeting AR (28)
miR-574	Regulating the testis development and reproduction by targeting AURKA (29)	miR-106a	Regulating the renewal and differentiation of spermatogonial stem cells by targeting STAT3 and Ccnd1 (30)
miR-140	testis differentiation (31)	miR-449a	Regulating the proliferation of spermatogonia by targeting CEP55 (32)
miR-125a	Regulating the testis degeneration by targeting SOD-1 (33)	miR-7	Regulating the differentiation of germ stem cells into primary spermatocytes by targeting Bam (34)
miR-202	male differentiation and development (35)	miR-135a	Regulating the proliferation and renewal of spermatogonial stem cells by targeting Foxo1 (36)
miR-215	Regulating the testis early developmental stage by targeting p53 (37)	miR-196a	Regulating the proliferation and apoptosis of immature sertoli cell by targeting RCC2 and ABCB9 (38)
		miR-10b	Regulating the proliferation of spermatogonial stem cells by targeting KLF4 (39)
		miR-21-5p	Regulating the renewal of spermatogonial stem cells by targeting ETV5 (40)
		miR-15a	Regulating the differentiation of spermatogonial stem cells by targeting Ccnt2 (41)
		miR-26a	Regulating the proliferation and promotes apoptosis of sertoli cells by targeting PAK2 (19)
		miR-140	Regulating the transformation from spermatogonia cells to primary spermatocytes (42)
		miR-202	(1) Regulating the proliferation, apoptosis, and synthesis of sertoli cells by targeting LRP6 and Cyclin D1 (43) (2) Regulating the renewal and differentiation of spermatogonial stem cells by targeting GDNF and RA (44)

### RNA-seq and miRNA-seq Data Validation

**Figure 6:**
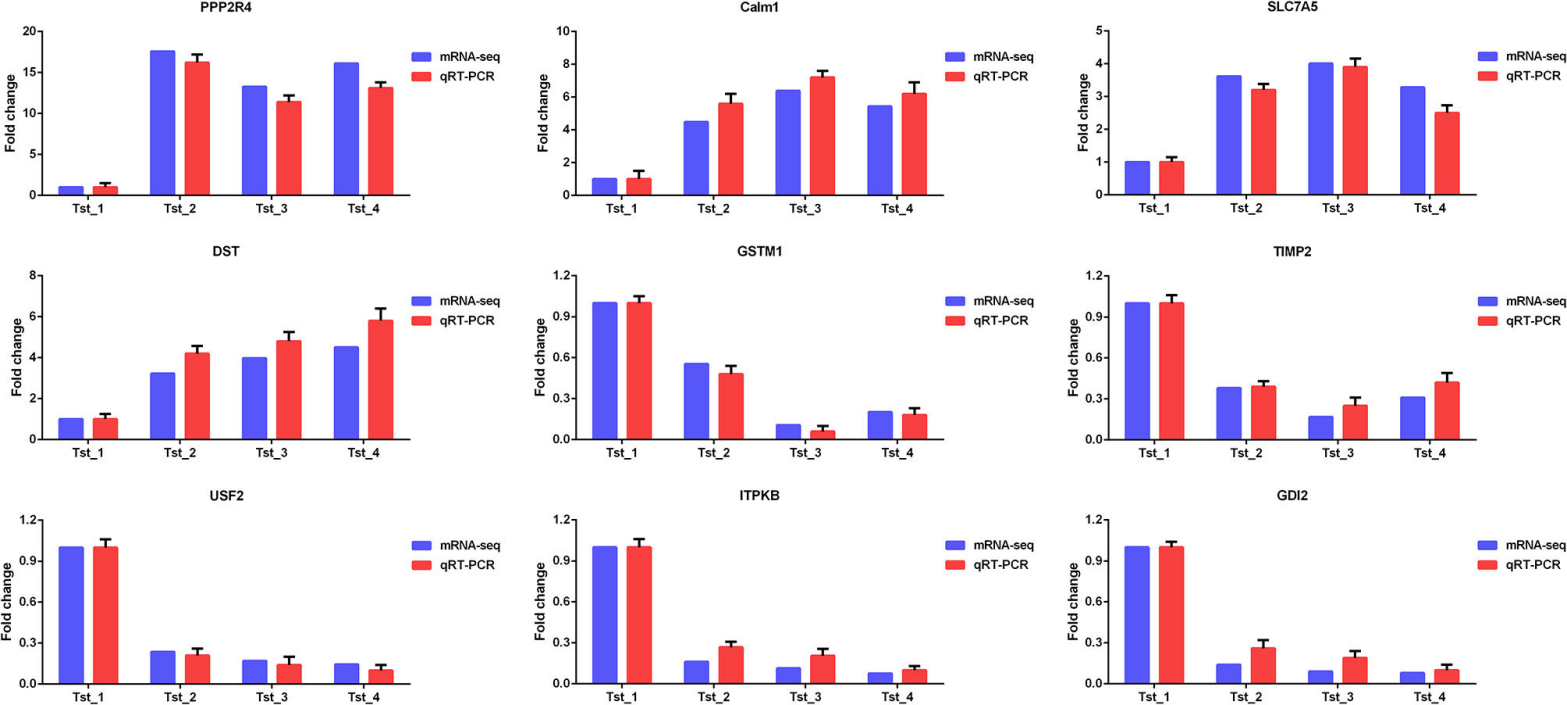
Q-PCR validation of mRNA-seq data.

**Figure 7:**
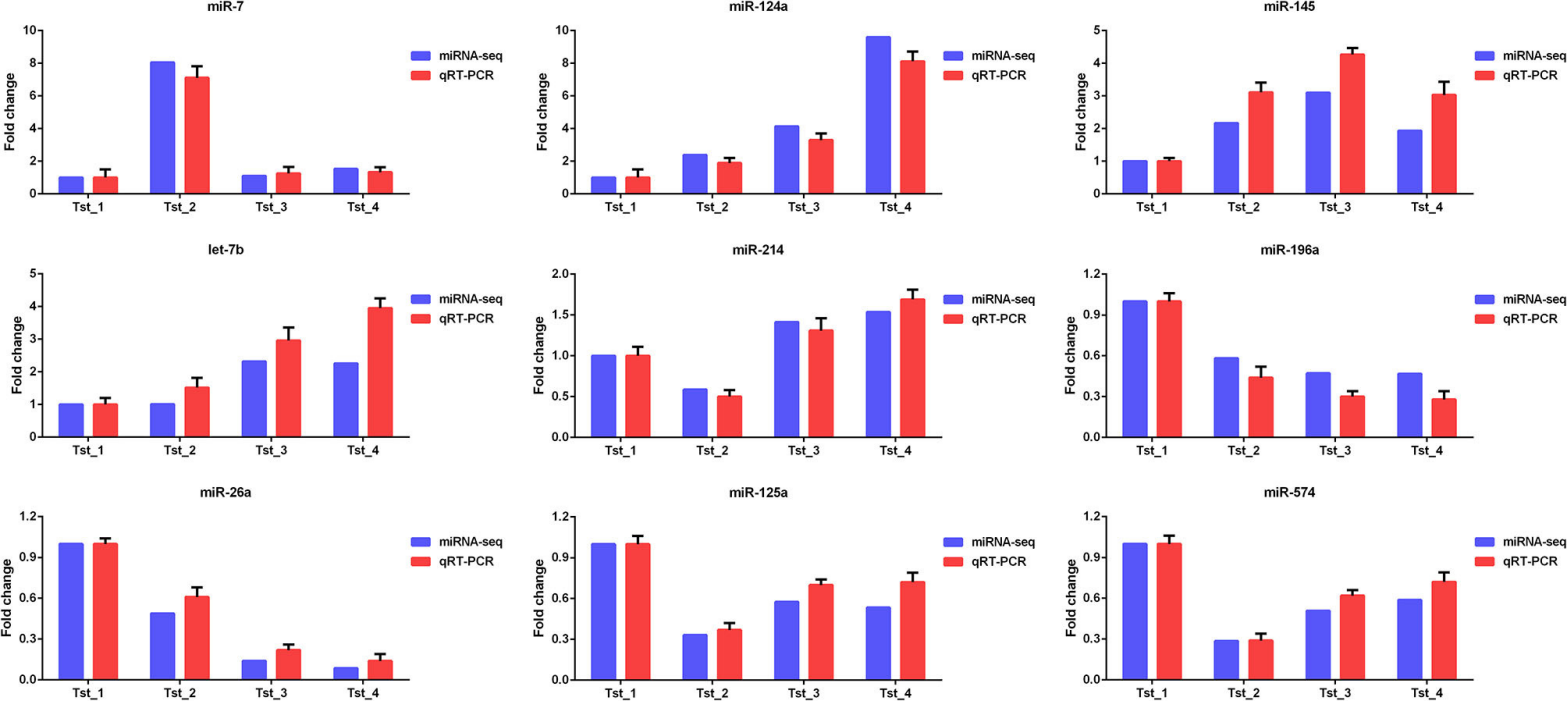
Q-PCR validation of miRNA-seq data.

Nine mRNAs (PPP2R4, Calm1, SLC7A5, DST, GSTM1, TIMP2, USF2, ITPKB, and GDI2) and nine known miRNAs (miR-7, miR-124a, miR-145, let-7b, miR-214, miR-196a, miR-26a, miR-125a, and miR-574) were randomly selected to verify the RNA-seq and miRNA-seq data by Q-PCR. As shown in Figures 6, 7, Q-PCR data were basically consistent with sequencing data in the four stages. The results indicated that our sequencing data were reliable, although the fold change was not completely consistent.

### miR-145 Targets IGF1R

**Figure 8:**
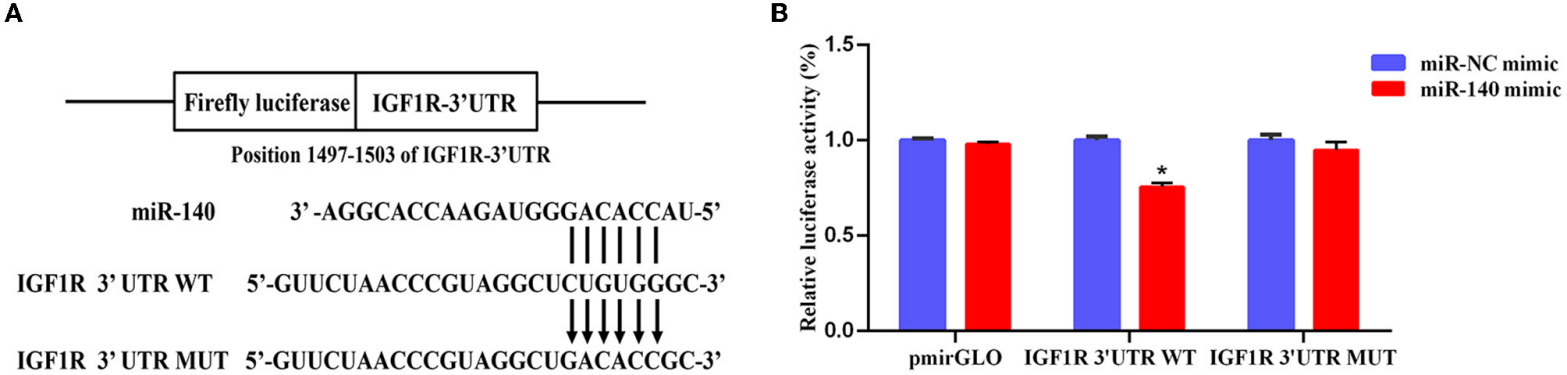
Detection of interactions between miR-140 and IGF1R by dual luciferase reporter system. **(A)** Binding site sequence of miR-140 and target gene IGF1R. **(B)** miR-140 mimic was cotransfected with IGF1R 3 'UTR WT or IGF1R 3 'UTR MUT to detect the luciferase activity for 48 h. *p < 0.05.

IGF1R was observed to be a major target of miR-140-5p based on bioinformatics databases. To verify their targeted regulatory relationship, we performed the Luciferase reporter assays and confirmed that, compared with the negative control, the luciferase activity of IGF1R receptor decreased by 75.6% after co-transfection of mir-140 mimics for 48 h. The results showed that miR-140 can directly target IGF1R-3′UTR (Figure 8).

## Discussion

The improvement of the reproductive efficiency of Sika Deer, especially in the male deer, was essential for livestock production. The testis development and spermatogenesis, which were mainly regulated by uniquely expressed genes at different developmental stages, were key factors affecting the reproductive efficiency of male deer. In this study, we analyzed mRNAs and miRNAs expression in the testes from 1-, 3-, 5-, and 10-year-old testis, which represent the juvenile, adolescence, adult, and aged stages, using high throughput deep sequencing. The aim of this study was to identify crucial miRNAs and mRNAs in testis development and spermatogenesis of Sika Deer. This analysis will help reveal biomarkers related to the reproductive efficiency of male Sika Deer.

In this study, the DE unigenes in Sika Deer testes at four development stages were enriched by GO. It was found that testicular development at different stages was regulated by different genes and biological functions, which was a complex biological process. In pathway analysis, for Tst_2 vs. Tst_1 contrast, the most significant pathway was enriched in cell adhesion molecules (CAMs), MAPK signaling pathway, insulin signaling pathway, and estrogen signaling pathway. These signal pathways were closely related to testicular development and spermatogenesis. For example, the pathway of CAMs can control the sexual and asexual development (45). MAPK signaling pathways were involved in mediating the mitogenic effect of IL-1 on Sertoli cells *in vitro* (46). Insulin signaling pathway played the essential role in regulating the final number of Sertoli cells, testis size, and daily sperm output (47). Estrogen signaling pathway had an important influence on the proliferation, apoptosis, survival, and maturation of sperm (48). It could be seen that these signaling pathways play an important role in the early stages of sexual maturity. Furthermore, Hedgehog signaling pathway was an overlapping pathway in Tst_2 vs. Tst_1 and Tst_3 vs. Tst_2. The Hedgehog signaling pathway has been reported to regulate the development of testicular cord and spermatogonial stem cells (49, 50). Phagosome was an overlapping pathway in Tst_3 vs. Tst_2 and Tst_4 vs. Tst_3. Phagosome was involved in the regulation of spermatogenesis (51). Glycerophospholipid metabolism was an overlapping pathway in three groups. It has been reported that glycerophospholipid metabolism was predicted to have dramatic effects on the male sexual differentiation and development (52). The results suggested that these pathways play a vital role in the testis development and spermatogenesis.

The core mRNA–mRNA network was constructed to reveal the regulatory relationship of testis development and spermatogenesis. In Tst_2 vs. Tst_1, the top DE unigenes interaction networks participated in MAPK signaling pathway, Wnt signaling pathway, oxytocin signaling pathway, and hedgehog signaling pathway. Among them, Wnt signaling pathway played a suppression role in mouse and human spermatogonia, which was a prerequisite for the normal development of primordial germ cells (53). The CTNNB1 and GSK3B were the core of this network. CTNNB1 could participate in the Wnt signaling pathway by encoding the β-catenin gene. Overexpression of CTNNB1 led to gender reversal, which was essential for male reproduction (54). GSK3B could precisely control the testis development of tambaqui by regulating Wnt signaling pathway and/or sox9 expression (55). Furthermore, the top DE unigene interaction networks participated in the adherens junction signaling pathway and calcium signaling pathway both in Tst_3 vs. Tst_2 and Tst_4 vs. Tst_3. Adhesive junctions mainly occur at the seminiferous epithelium (Sertoli–Sertoli and Sertoli–germ cell interfaces), so that the developing germ cells can migrate from the basal compartment of the seminiferous epithelium toward the adluminal compartment for further development (56). CTNNA1 and CTNNB1 were the core members of the Adherens junction signaling pathway. As we all know, CTNNA1 was a member of the catenin protein family, which was crucial for cell adhesion. It was located on the plasma membrane and binds to cadherin (57). In addition, calcium signaling pathway was necessary for many physiological processes such as spermatogenesis, sperm motility, capacitation, acrosome reaction, and fertilization (58). CDH2 was an important member of calcium signaling pathway. The lack of Cdh2 in Sertoli cells caused damage to the blood–testis barrier, which in turn led to meiosis and spermatogenesis failure (59). Taken together, through interaction network analysis of DE unigenes, we have screened the core regulatory unigenes for Sika Deer testis development and spermatogenesis, such as CTNNA1, CTNNB1, GSK3B, and CDH2.

It was known that miRNA played an important regulatory role in testis development and spermatogenesis, but there was no related research on its regulation of Sika Deer testis. We integrated the changes of miRNA profiles in testis at four different developmental stages. The ranges of 20–24nt small RNAs were the main size of the population in Tst_1, while Tst_2, Tst_3, and Tst_4 were mainly 25–32 nt in size. This result suggested that reads longer than 25 nt were mainly from piRNA. As a newly discovered small regulatory RNA, piRNA was abundantly enriched in mature testis (60). In our research, the expression profile of miRNAs had obvious growth stage specificity, in which miRNAs associated with testis development and spermatogenesis were expressed at specific growth stages. For example, miR-106b, miR-19b, miR-27a-3p, miR-27b, miR-31, and miR-335 were expressed specifically on the juvenile stage (61–66); miR-449a was expressed specifically on the adolescence stage (32); miR-296-3p was expressed specifically on the adult stage (67). The results suggested that the growth period-specific miRNAs have an important influence on testis development and spermatogenesis.

This study tried for the first time to determine the key miRNA targets by integrated analysis and the expression profiles of miRNA and mRNA in the testes of Sika Deer. In Figure 4A, miR-7 and miR-145 related to testis development were upregulated. The target mRNAs of these miRNAs included HMGB1, HSPA8, CYP11A1, ALKBH5, etc., as the central node genes. Among them, HMGB1, as a paracrine host defense factor in the testis, was translocated from testicular cells and blocking its action by ethyl pyruvate regulated inflammatory reactions in testes and spermatogenic damage (68, 69). Testis-specific HSPA8 gene was confirmed to decrease with the development of goat testis, which may be due to dilution by the maturing germ cell population (70). Lower concentrations of HSPA8 were associated with subfertility in men (71). CYP11A1, as a steroidogenic enzyme gene, induced gonadal differentiation and development by regulating steroidogenesis (72). CYP11A1 was fetal Leydig cell marker genes (73). Kim et al. found that CYP11A1 was responsible for C21-steroid hormone metabolism and played an important role in the recovery of testicular aging in rats (74). Baker et al. found that there was a significant correlation between the rate of ALKBH5 protein-coding substitutions and the rate of testis size evolution. ALKBH5 drives the evolution of testis size in tetrapod vertebrates (75). In our study, with the development of Sika Deer testes, upregulation of miR-7 and miR-145 inhibited the expression of HMGB1, HSPA8, CYP11A1, and ALKBH5, respectively, thereby regulating testicular maturation.

In Figure 4B, miR-26a, miR-125a, miR-140, miR-202, miR-215, and miR-574 related to testis development were downregulated. The target mRNAs of these miRNAs included IGF1R, Piwil, StAR, TSPYL1, etc., as the central node genes. Among them, IGF1R was required for the appearance of male gonads and thus for male sexual differentiation (76). IGF1R has also been confirmed to decrease in expression with increasing age in goat testes, presumably due to maturation of cells and cessation of testis growth (70). Piwil mainly appeared in the early stage of gonadal development, and its expression in testis first increased and then decreased. It was an important regulatory gene of germ cell division during gonadal development (77). StAR was mainly expressed in Leydig cells of mammalian testis and played an imperative role in testosterone biosynthesis and male fertility (78). The interstitial lipid deposits worsened considerably in StAR knockout mice testes, and the germ cells showed the histological characteristics of apoptosis, which was consistent with the unsatisfactory androgen secretion (79). TSPYL1 specifically recruited ZFP106 through amino acids 412–781, which was considered to be a key pathway involved in testes development (80). It was first reported in 2004 that the loss of function mutations of TSPYL1 gene caused Sudden Infant Death with Dysgenesis of the Testes syndrome (81). Taken together, it was speculated that the downregulation of miR-140 promoted the expression of the IGF1R, Piwil, and TSPYL1 during the development of Sika Deer testes, thereby promoting testicular maturation. In conclusion, these DE miRNAs might play an important role in the regulation of the development of Sika Deer testes through target genes.

In Figure 5A, let-7b, miR-214, miR-124a, miR-106a, miR-449a, and miR-7 related to spermatogenesis were upregulated. The target mRNAs of these miRNAs included HIF1A, CSF1, TCP11, SPAG17, PEBP1, AKAP3, CNOT7, PHB, etc., as the central node genes. Among them, the upregulation of miR-7 inhibited the expression of target genes HIF1A and CSF1. HIF1 was a highly specific nuclear transcription factor, which was closely related to the apoptosis of spermatogenic cells (82). After silencing the HIF1A gene in the testis of varicocele rats, the apoptosis of spermatogenic cells was reduced and the spermatogenic function of the testes was significantly improved (83). CSF1 was an extrinsic stimulator of spermatogonial stem cells self-renewal (84). The protein level of CSF1 was the highest in the testis of 1-week-old mice and decreased significantly with age (2–12 weeks). CSF1 was also involved in inducing the proliferation and differentiation of spermatogonial cells to meiotic and postmeiotic stages (85). Therefore, it was speculated that the upregulated miR-7 promoted spermatogenesis through target genes HIF1A and CSF1. Similarly, the upregulation of miR-214 inhibited the expression of target genes TCP11, SPAG17, PEBP1, AKAP3, CNOT7, and PHB. TCP11 was a testis-specific gene, which played a role in elongated spermatids to confer proper motility in mature sperm (86). In addition to affecting sperm motility, SPAG17-deficient mice were sterile due to prevention of the normal manchette structure, protein transport, and formation of the sperm head and flagellum (87). PEBP1 was specifically expressed in the head of the elongated spermatids and mature spermatozoa. PEBP1 could affect the function of mature sperm in pachytene primary spermatocytes and spermatids by activating the expression of ERK1/2 (88). AKAP3 was one of the main components of sperm tail fibrous sheath formed during spermatogenesis. In the elongating stage of mice spermiogenesis, the protein complexes of AKAP3, PKA, and RNA binding proteins can be synthesized under the regulation of PKA signaling to participate in the process of spermatogenesis (89). CNOT7 was a CCR4-related transcription cofactor, which is essential for spermatogenesis. The maturation of spermatids in seminiferous tubules of CNOT7 deficient mice was asynchronous and damaged (90). PHB was mainly located in mitochondria during spermatogenesis, which regulated the proliferation of spermatogonia, mitochondrial morphology, and function in spermatogenic cells (91). PHB-deficient mice resulted in infertility due to meiotic pachytene arrest, mitochondrial morphology, and function impairment (92). In conclusion, it was speculated that upregulated miR-214 was involved in spermatogenesis through target genes TCP11, SPAG17, PEBP1, AKAP3, CNOT7, and PHB.

In Figure 5B, miR-135a, miR-196a, miR-10b, miR-21-5p, miR-15a, miR-26a, miR-140, and miR-202 were downregulated. The target mRNAs of these miRNAs included DPY19L2, HSPA4L, PELO, Piwil1, TSGA10, IP6K1, MNS1, BRDT, CEP55, SMC6, etc., as the central node genes. Among them, Piwil1 was crucial for spermatogenesis because it used different domains to interact with several spermiogenic mRNAs after meiosis, and partially participated in translation regulation through 3′-UTRs (93). TSGA10-deficient mice had significantly reduced sperm motility due to the disorder of mitochondrial sheath formation during spermatogenesis (94). The spermatids of IP6K1-deficient mice express TNP2 and PRM2 prematurely, resulting in abnormal elongation of spermatid and inability to complete spermatid differentiation (95). MNS1 was located in the sperm flagella and played an important role in spermatogenesis, the assembly of sperm flagella, and ciliary movement (96). Thus, the downregulated miR-140 promoted spermatogenesis through target genes Piwil1, TSGA10, IP6K1, and MNS1. Similarly, BRDT was a key regulator of transcription in meiotic and post-meiotic cells. The loss of BRDT function destroyed the epigenetic state of meiotic sex chromosome inactivation in spermatocytes (97). CEP55 gene silencing could inhibit the proliferation of mouse spermatogonia and play a key role in spermatogenesis (98). SMC6 played a role in preventing aberrant recombination events between pericentromeric regions in the first meiotic prophase (99). These findings suggested that miR-26a low-expression partially regulated spermatogenesis by promoting the expression of BRDT, CEP55, and SMC6. In addition, the defect of DPY19L2 gene was the main genetic cause of human globozoospermia, which may be related to the defect of chromatin compaction during spermatogenesis and sperm DNA damage (100). HSPA4L was highly expressed from late pachytene spermatocytes to postmeiotic spermatids. The germ cells of HSPA4L deficient mice were apoptotic, resulting in a decrease in the number of sperm count (101). PELO gene mutation led to cell cycle arrest in the G2/M transition period before the first meiosis during spermatogenesis (102). DPY19L2 was the target gene for miR-135a, miR-202, miR-196a, miR-21-5p, and miR-140. HSPA4L was the target gene for miR-21-5p, miR-26a, miR-135a, miR-140, and miR-202. PELO was the target gene for miR-196a, miR-21-5p, miR-26a, and miR-140. It was speculated that these miRNAs might promote spermatogenesis by upregulating the target genes DPY19L2, HSPA4L, and PELO.

In addition, IGF1R was a widely expressed tyrosine kinase that regulated cell proliferation, differentiation, and survival (103). As one of the sex differentiation genes, IGF1R played an important role in regulating Sertoli cell number, testis size, and daily sperm output (47). Pintus et al. found that the Sertoli cell number affected testis size, sperm quantity, and sperm quality in red deer (104). It could be seen that IGF1R was crucial to regulate testis development in Cervidae. Bioinformatics analysis and dual luciferase reporter analysis showed that the 3′UTR of IGF1R matched the seed sequence of miR-140. miR-140 could reduce the expression of IGF1R. However, the hypothesis that miR-140 is involved in Sika Deer testis development and spermatogenesis by targeting IGF1R needs to be further verified.

## Conclusion

This study first reported the important miRNA–mRNA network derived by the global analysis and integration of the changes of miRNA and mRNA levels in Sika Deer testis. Some mRNAs and miRNAs were found to be common, which means that they could be necessary for testis development and spermatogenesis. At the same time, some differential expressions of mRNAs and miRNAs were found in testis at different developmental stages (1-, 3-, 5-, and 10-year-old), which means that they could play different but important roles in testis development and spermatogenesis. In particular, the binding sites of miR-140 with IGF1R was validated. In the future, we will focus on studying the role of individual miRNA in testicular development and exploring relevant pathways to reveal the mechanism of Sika Deer spermatogenesis.

## Data Availability Statement

The datasets presented in this study can be found in online repositories. The names of the repository/repositories and accession number(s) can be found in the article/Supplementary Material.

## Ethics Statement

The animal study was reviewed and approved by Jilin Agricultural University Committee on the use of live animals. Written informed consent was obtained from the owners for the participation of their animals in this study.

## Author Contributions

BJ, RD, and ZC: conceptualization. BJ, LZ, FM, XW, and FY: methodology. BJ, JL, ND, XL, YZ, and QG: software. BJ, KS, and FZ: writing—original draft preparation. BJ, FL, and RC: writing—reviewing and editing. RD and ZC: supervision. All authors contributed to the article and approved the submitted version.

## Funding

This study was funded by National Natural Science Foundation of China (Grant/Award Nos. 32002171 and 81802869) and Science and Technology Research Project of Jilin Province Education Department (Grant/Award No. JJKH20220364KJ).

## Conflict of Interest

The authors declare that the research was conducted in the absence of any commercial or financial relationships that could be construed as a potential conflict of interest.

## Publisher's Note

All claims expressed in this article are solely those of the authors and do not necessarily represent those of their affiliated organizations, or those of the publisher, the editors and the reviewers. Any product that may be evaluated in this article, or claim that may be made by its manufacturer, is not guaranteed or endorsed by the publisher.

## Supplementary Material

Supplementary Figure 1 The top 20 GO categories of DE unigenes in each comparable group.

Supplementary Figure 2 The top 20 enriched KEGG pathways of DE unigenes in each comparable group.

Supplementary Figure 3 Characterization of miRNA-seq mapped reads from the small RNA sequencing of testis.

Supplementary Figure 4 The top 20 enriched GO terms for the potential genes targeted by DE miRNAs.

Supplementary Figure 5 The top 20 enriched KEGG pathways for the potential genes targeted by DE miRNAs.

Supplementary File 1 Summary of transcriptome data.

Supplementary File 2 FPKM statistical of testis transcriptome in Sika Deer.

Supplementary File 3 The lists of DE unigenes from RNA sequencing.

Supplementary File 4 GO categories of differential unigenes in four growth periods.

Supplementary File 5 KEGG pathways of differential unigenes in four growth periods.

Supplementary File 6 Interaction networks of DE unigenes in each comparable group.

Supplementary File 7 Summary of miRNAome data.

Supplementary File 8 Statistical information of small RNAs mapping to reference sequence.

Supplementary File 9 Small RNAs annotation.

Supplementary File 10 Identification of known miRNAs and novel miRNAs.

Supplementary File 11 The lists of DE known miRNAs.
