# Supplementary figures and images for: Comparison of miRNA and mRNA Expression in Sika Deer Testes With Age
Source: Front Vet Sci. 2022 Apr 5;9:854503. doi: 10.3389/fvets.2022.854503 (PMC9019638; doi:10.3389/fvets.2022.854503)

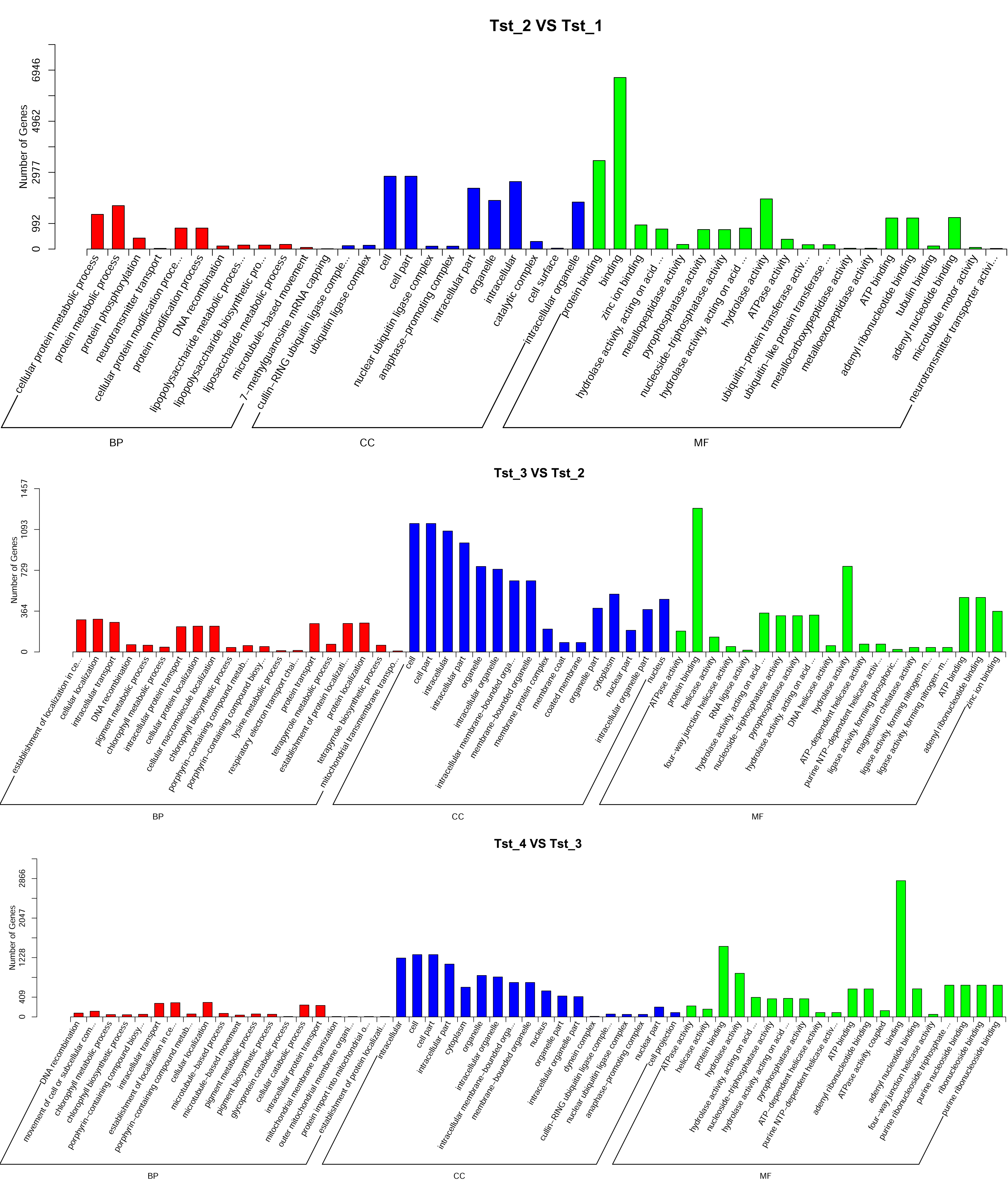

Supplement: Supplementary Figure 1 — The top 20 GO categories of DE unigenes in each comparable group. [file Image_1.TIF]

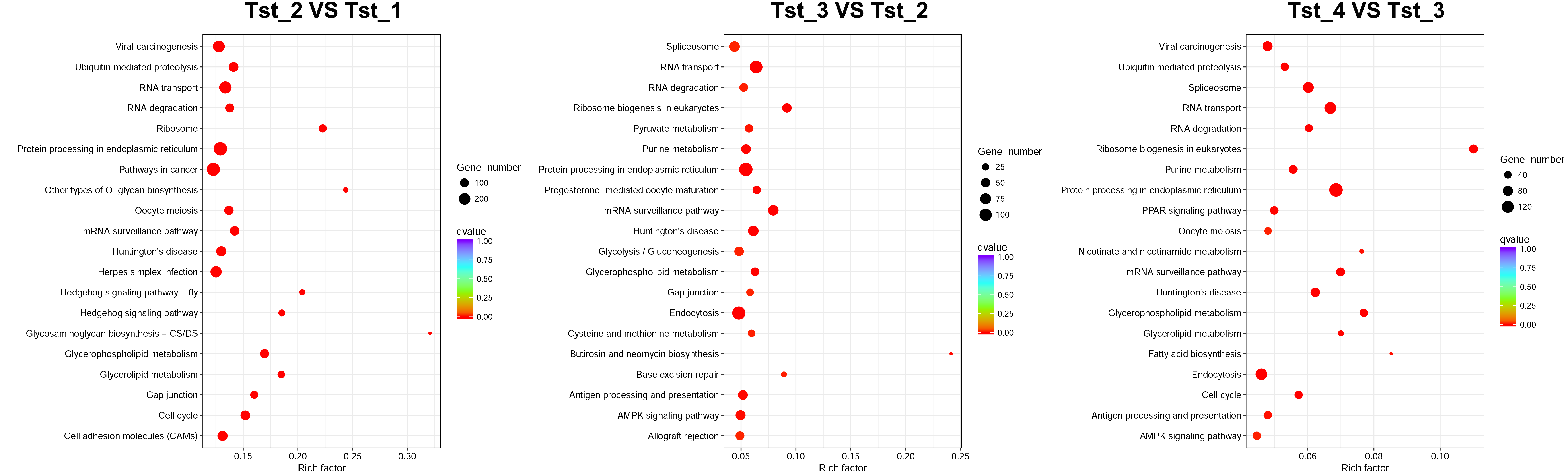

Supplement: Supplementary Figure 2 — The top 20 enriched KEGG pathways of DE unigenes in each comparable group. [file Image_2.TIF]

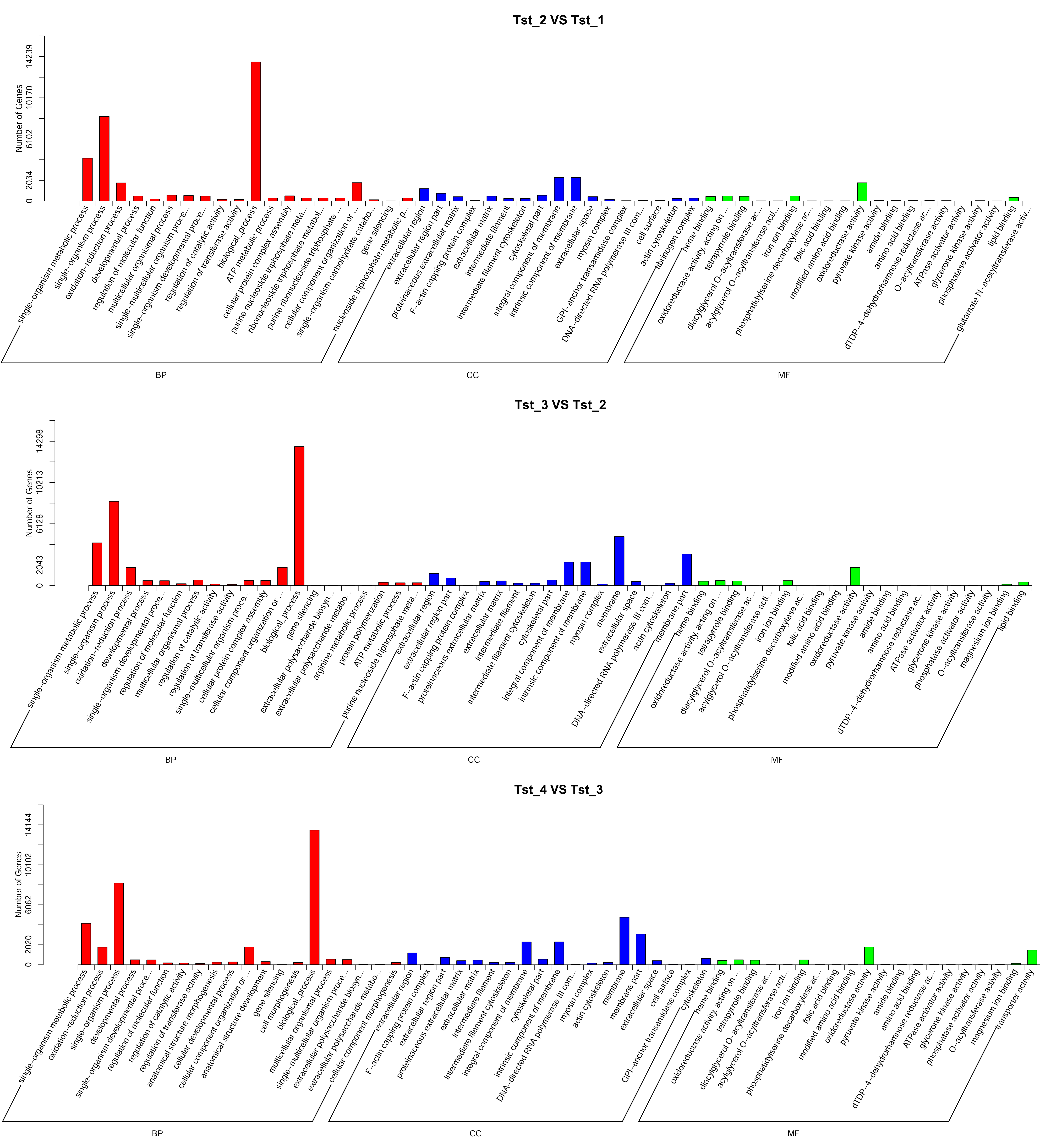

Supplement: Supplementary Figure 4 — The top 20 enriched GO terms for the potential genes targeted by DE miRNAs. [file Image_4.TIF]

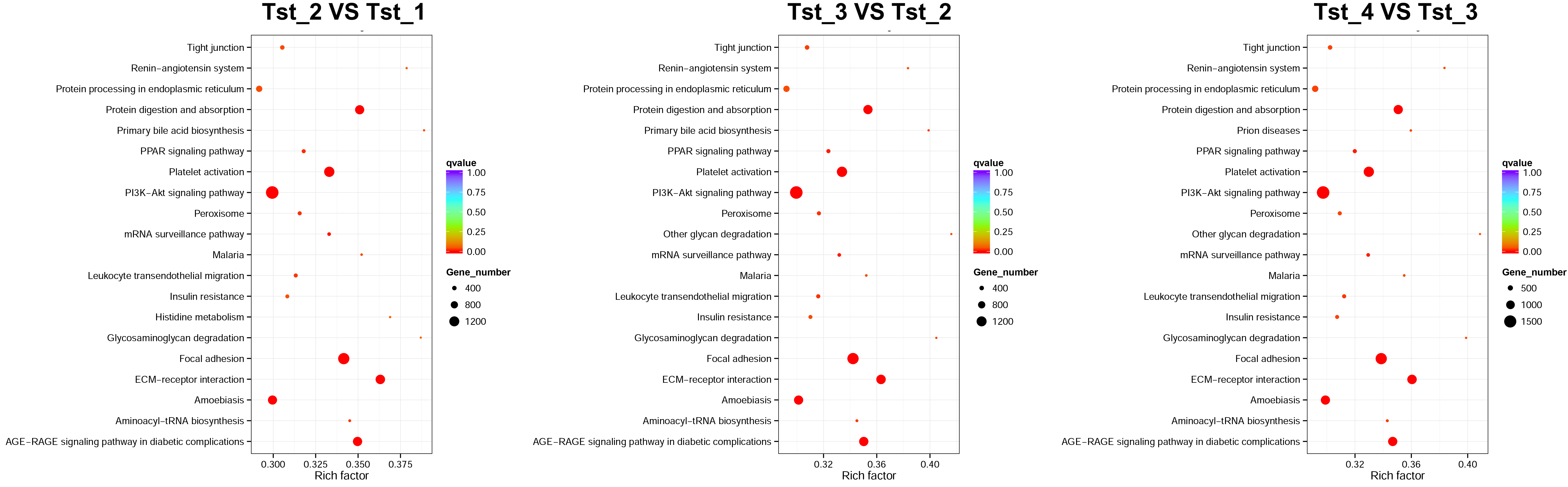

Supplement: Supplementary Figure 5 — The top 20 enriched KEGG pathways for the potential genes targeted by DE miRNAs. [file Image_5.TIF]
